# Robotic Technologies for Hand Recovery After Stroke: A Reflection on Research Paths in Honor of Dr. Steven Wolf

**DOI:** 10.1177/15459683261448473

**Published:** 2026-05-29

**Authors:** David J. Reinkensmeyer

**Affiliations:** 1 Department of Mechanical and Aerospace Engineering, University of California at Irvine, USA; 2 Department of Anatomy and Neurobiology, University of California at Irvine, USA; 3 Department of Biomedical Engineering, University of California at Irvine, USA; 4 Department of Physical Medicine and Rehabilitation, University of California at Irvine, USA

**Keywords:** robotics, rehabilitation, stroke, movement, proprioception, technology, training

## Abstract

Over the past 35 years, my work has focused on developing and studying robotic technologies to promote hand and arm recovery after stroke. In this Point-of-View, written for a special issue honoring Steven L. Wolf, I reflect on the personal and theoretical factors that shaped my research path, and how they intersect with Steve’s pioneering contributions to stroke rehabilitation. I first provide a brief overview of the personal factors that influenced my research journey. Then, I turn to theoretical factors, highlighting conceptual synergies between Constraint-Induced Movement Therapy (CIMT) and robot-assisted therapy, including the principles of dose, task-specific training, shaping, prevention of slacking, participant selection, and the critical role of proprioception. Finally, I discuss 5 implications for future directions in robotic therapy consistent with Dr. Wolf’s vision of intensive, patient-centered, and mechanistically grounded therapy: (1) the use of shared principles from CIMT and robot-assisted therapy to guide the design of advanced rehabilitation technologies; (2) precision rehabilitation that prioritizes proprioception; (3) the design of practical, widely adoptable robotic systems; (4) the development of real-time biomarkers and closed-loop training systems that optimize recovery; and (5) the need for deeper collaboration with people with lived experience of disability to address unsolved challenges. Steve’s impactful results, driven by his curiosity, rigor, and openness to diverse approaches, have profoundly influenced my career, the ideas I present here, and the broader field of robotic therapy.

## Introduction

I first became familiar with Dr. Steven Wolf’s work during my early years in rehabilitation engineering research, when his work on Constraint-Induced Movement Therapy (CIMT) began to reshape expectations for recovery after stroke. I later had the opportunity to interact with Steve at conferences and through scientific exchanges and was struck by his intellectual curiosity, openness to new ideas (including robot-assisted training), and willingness to engage across disciplinary boundaries. In this perspective, at the request of the special issue organizers, I first reflect on the personal factors that have shaped my journey in rehabilitation robotics. I then detail theoretical considerations that have shaped my research approach, framing them in light of one of Steve’s key contributions, the EXCITE trial of CIMT. I conclude by discussing implications for future directions in neurorehabilitation technology suggested by Steve’s work and robotic therapy research.

## My Research Journey in Rehabilitation Robotics

The personal factors that influenced by research path began early: I wanted to be an inventor following an invention competition organized by my third grade teacher, Ms. Carol Dewoskian, in Wichita, Kansas. In junior and senior high school, I enjoyed making mechanically articulated wooden toys that mimicked human and animal movement, as well as board and computer games. Then, as an undergraduate at MIT, I worked with Chris Atkeson on a project that used the Connection Machine – a massively parallel computer – and memory-based learning to simulate learning-based control of throwing robots and Marc Raibert’s hopping robots.^1^ That experience showed me how robots can emulate biological movement and how biologically relevant learning algorithms can be analyzed at the computational and algorithm levels of analysis, not just the hardware levels, as Marr suggested in his framework for understanding information processing systems,^2^ which has strongly shaped my approach to neuroscientific questions in neurorehabilitation. At the same time, my favorite undergraduate class was part of the innovative 6-1-b major at MIT, Bioelectrical Engineering, a precursor of biomedical engineering. 6.023 J Quantitative Physiology: Sensory and Motor Systems focused on “studies of sensory and motor physiology with objectives of establishing quantitative models.”^3^ Through this class and my undergraduate research project I fell in love with the human motor control system and robotic design. Yet, I still desired to be an inventor who created technologies that directly helped people.

**Figure 1.**
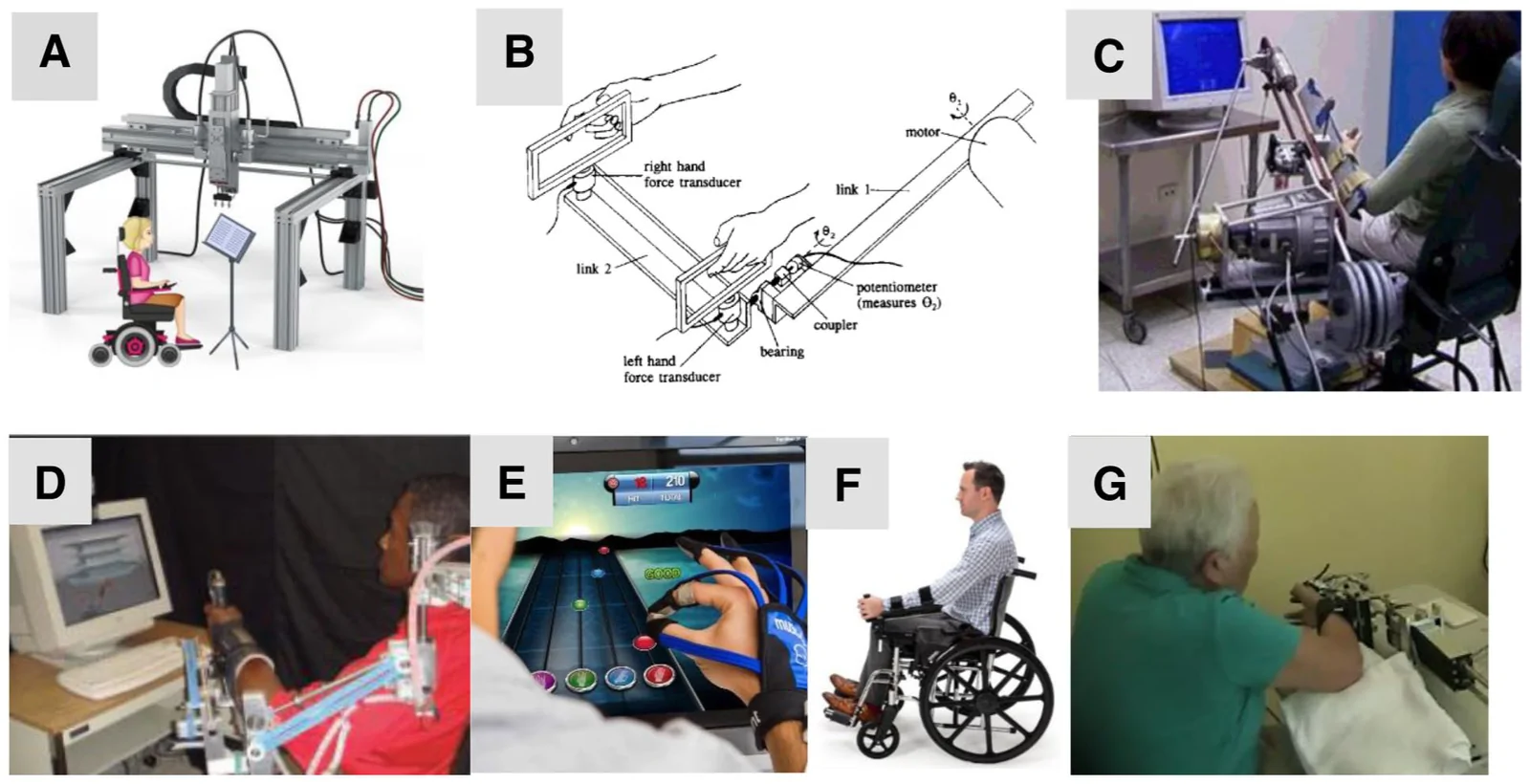
Robotic rehabilitation devices mentioned in this Perspective. (A) As a graduate student in robotics, I programmed a large, industrial robot to turn pages in a book for a friend with cerebral palsy. (B) The bimanual lifting rehabilitator, developed when I was a graduate student, one was of the first robotic therapy devices and focused on providing task-specific, assistance-as-needed. Reproduced from^4^ with permission. (C) I built the ARM Guide as a postdoctoral fellow, developing it as a tool for mapping workspace deficits and for assisting in reaching practice after stroke.^5^ It used a trombone-like, linear guide mounted on a lockable swivel to assist in reaching movements to different targets in three-dimensional space. (D) An early project in my laboratory at UC Irvine was T-WREX,^6,7^ a non-robotic, spring-driven arm support that was commercialized as ArmeoSpring. The idea of T-WREX was to test if similar therapeutic benefits could be obtained using a passive (ie, spring-driven) device compared to what had already been obtained with robotic devices. T-WREX also integrated a hand sensor that could detect trace amounts of grip force, allowing us to integrate coordinated arm/hand actions in task-oriented games like shopping or cooking. (E) A graduate student in my lab, Nizan Friedman, realized that we could use popular video games for rehabilitation training, resulting in the MusicGlove,^8^ a sensorized glove for intensive grip practice using a musical computer game similar to GuitarHero. Reproduced from^74^ with permission. (F) The FINGER robot,^9^ built by Eric Wolbrecht’s group at University of Idaho, has been a pivotal tool we’ve used to test the role of finger proprioception in hand movement training.^10^ (G) A recent project focused on making training highly accessible resulted in Boost, a dynamic arm support for practicing elbow extension during wheelchair time.^11^

Moving to graduate school at Berkeley, I joined a campus club and soon met a graduate student with severe cerebral palsy who announced that she needed help cooking and eating on Friday nights before the club met. I ended up volunteering and becoming close friends with her. I had custom-designed my own graduate curriculum for my PhD program in Electrical Engineering that blended robotics with neuroscience. A robotics course I was taking required me to complete a final project. I asked my friend if there were any tasks she thought a robot could help her with. She said turning pages in a book. I programmed at $100K cartesian industrial robot with a 100 ft^2^ footprint to turn pages in a book. I got it to work, but it was completely impractical (Figure 1(A)).

At the same time, I realized that my friend required regular physical therapy but used little in the way of technology to complete her therapy, relying on one-on-one interactions with a therapist at a gym. I had the idea of using robotic devices to assist in semi-autonomous physical therapy, an idea that had not yet been pursued vigorously, although Neville Hogan had just initiated work on the first robotic therapy device, MIT-MANUS, via an National Science Foundation grant starting in 1989.^12^ I had the good fortune of being supported by an NSF graduate fellowship, allowing me freedom in choosing my research topic, and the additional good fortune of being mentored by muscle physiologist Steve Lehman, who was remarkably open and encouraging to this new direction. A few robots were being developed to assist human movement at this time, around 1990, with key work by Homayoon Kazerooni, a member of my dissertation committee. Kazerooni had developed some of the first “human power amplifiers” (or “extenders”) for human augmentation that were important precursors to legged exoskeletons for people with spinal cord injury.^13^ Yet, movement training robots that were based on the idea that human-robot physical interactions could be leveraged to shape use-dependent plasticity were unexplored.

We had been studying bimanual control in the Lehman lab, including an early form of a robot-applied force field that produced a bimanual after effect,^14^ and, together with my lab mate Pete Lum – who later went on to make important contributions to rehabilitation robotics including building a system to automate CIMT^15-17^ – we decided to build robotic devices for bimanual movement training^4,18^ (Figure 1(B)). We drew on the rather limited literature that suggested bimanual training had potential after stroke. We didn’t fully know what we were doing – a situation captured by a line sometimes attributed to Einstein that my graduate students and I often appreciate: “If we knew what we were doing, it wouldn’t be called research.”

We had few resources to clinically test our bimanual rehabilitation robots. To simulate stroke, we occluded blood flow in our arms with a cuff, temporarily inducing hemiparesis.^18^ Our first grant application was brutally rejected by reviewers at NIH. However, we saw potential and enjoyed brainstorming about the potential of “rehabilitators” (a term we proposed^19^ that mostly did not stick, except see: Britannica^20^). Besides helping develop and test 2 bimanual training robots for my dissertation, I identified several theoretical problems for robotic rehabilitation that are still relevant, including whether they should promote “normal” movement, how they should adapt assistance, and ways in which they could infer the movement intent of the patient in order to assist intelligently.^19^

I applied for a postdoctoral fellowship at the Rehabilitation Institute of Chicago with Zev Rymer, who also saw the potential for robotic rehabilitation. Zev pushed me to think mechanistically about what robots might do in stroke movement rehabilitation, an approach I continue to emphasize in my laboratory. Specifically, I continue to envision our work as a cycle in which mechanistic experiments inform device design, and new devices enable the next scientific experiment. Within this cycle, we spin off devices for commercialization if end-users see them as useful and desirable. I also gained machine-shop experience, building the Arm Guide^5^ (Figure 1(C)), and clinical experience, testing the Arm Guide with a cohort of individuals post-stroke as well as being exposed to the excellent work on spasticity, abnormal synergies, and motor adaptation being done at the Rehabilitation Institute of Chicago.

Around this time, the first positive results in small clinical trials testing CIMT were emerging,^21^ suggesting the importance of high-dose movement training. In addition, seminal work by Nudo et al^22^ demonstrated that rehabilitative training of the hand after cortical injury can drive reorganization of motor cortex representations and support functional recovery. Together, these advances gave me the sense that movement rehabilitation science was beginning to make a stand as a rigorous, mechanistically grounded field, but it was obvious it needed better engineering tools, at a minimum to better quantify movement recovery and training, but also, potentially to help deliver more training in a practical way. I applied for and won a National Institute on Disability and Rehabilitation Research field-initiated grant for robotic quantification of the workspace, an accomplishment I was so happy about that I remember skipping with joy as I got off the bus in Rogers Park to walk home to my new wife, who was studying Occupational Therapy at Rush University, and who had played an essential role in brainstorming, reality checks, and encouragement, back then and continuing throughout my career.

After applying to dozens of assistant professor positions and being rejected by nearly all of them, I had the incredibly good luck of being offered an assistant professor position at U.C. Irvine in Mechanical and Aerospace Engineering where I have now worked for the past 28 years. The combined challenge of inventing innovative robots and understanding neural recovery has been a fulfilling career. I am proud that 2 rehabilitation technologies my lab invented – T-WREX^6,7^ (Figure 1(D), commercialized as ArmeoSpring) and the MusicGlove^8,23^ (Figure 1(E)) – have logged millions of practice movements achieved by tens of thousands of individuals using them as commercial, medical devices. This work has been and continues to be significantly influenced by Steve Wolf’s work.

## Theoretical Factors Influencing Research in Robotic Therapy

**Figure 2.**
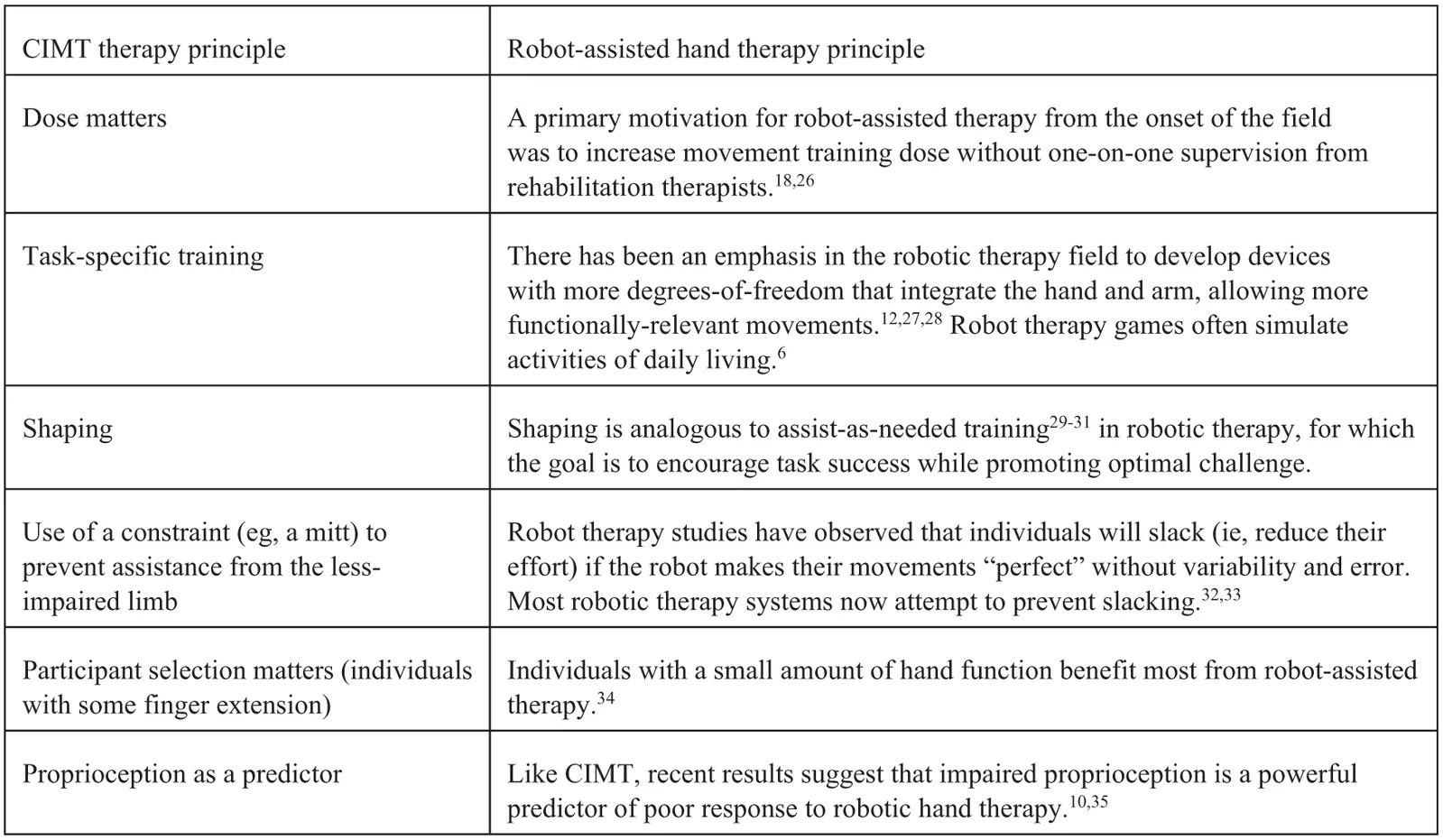
Conceptual synergies between constraint-induced movement therapy (CIMT) and robot-assisted therapy.

Steve Wolf is well known as the lead-author of one of the most important studies in stroke rehabilitation history: the EXCITE trial of Constraint-Induced Movement Therapy (CIMT), published in 2006.^24^ This trial was the first to demonstrate at scale with rigor that movement training can make a substantial difference in stroke hand recovery.^25^ It also made several important conceptual contributions that shaped and reinforced my own work and the entire field of rehabilitation robotics, 6 of which I highlight here (Figure 2).

CIMT and robotic therapy emerged during a similar period and, from the outset, both emphasized the need for high-dose training. While each was influenced by parallel developments in rehabilitation science making it difficult to establish direct causality, the principles established and validated at scale in the EXCITE trial were, in my view, highly influential in shaping the development of robotic therapy and continue to shape it.

### Dose Matters

In the EXCITE study, participants trained for up to 6 hours a day and wore the mitt during 90% of waking hours. After reflecting on the dose-response curve from a meta-analysis of rodent forelimb rehabilitation^36^ as well as dose-focused clinical trials of upper extremity stroke rehabilitation,^37-40^ I have come to believe that it is a cruel fate of nature that the amount of movement practice required to stimulate sufficient plasticity to make a more-than-modest effect on stroke hand recovery is all but impossible in the current rehabilitation landscape. Not many can train like Olympic athletes – or EXCITE trial participants. I see robotics technologies as a tool to push closer toward aspirational levels of training. Robotics technologies can be used semi-autonomously, working in concert with clinicians to supplement prescribed training. They can also adapt intelligently to impairment, and incorporate motivational features that are essential for long-term, intense engagement in rehabilitation. These motivational features include physical assistance, which is a means to reconnect volition to movement,^41^ thereby promoting movement success and enhancing motivation, self-efficacy, and perceived competence in quantifiable ways, as we have shown.^10,42^ Steve Wolf’s curiosity and willingness to experiment with a wide range of technologies to promote training intensity is impressive and influential: even after the success of the EXCITE Trial, he conducted studies on biofeedback with wearable sensors and EMG,^43^ wearable vibrotactile stimulation,^44^ vagus nerve stimulation,^45^ and robotic rehabilitation.^46^ Of robotic rehabilitation, he wrote in 1 paper:

Robotic neurorehabilitation has the potential to have a greater impact on impairment because of ease of deployment, applicability across a wide range of motor impairments, high measurement reliability, and the capacity to potentially deliver the optimal dose and intensity of training protocols that are patient specific.^46^

Another example of the conceptual synergy between CIMT and robotics is the fact that Pete Lum, my lab mate back at U.C. Berkeley, developed an automated system for providing CIMT called Automated Constraint-Induced Therapy Extension.^16^

### Task-Specific Training

Second, the EXCITE trial suggested that task-specific training is important. Participants practiced manual tasks that had relevance for their daily life. This has caused me and others to try to make robotic training more functionally-oriented by adding degrees of freedom and building games modeled after activities of daily living.^12,27,28^ However, while task specific training is effective, we and others have also found that simpler forms of machine-mediated training can have comparable effects.^12,27,28^ Task-specific training remains a de facto design goal in robotic therapy, but understanding the benefits and limitations of task-specific training for robotic therapy is an important, unresolved challenge.

### Shaping

Third, the EXCITE trial incorporated the idea of shaping, which refers to progressively titrating challenge to the ability of the patient. This is also the goal of the assist-as-needed, robotic training approach we have developed and promoted in our own work,^29,30,32^ similar to the performance-based progressive challenge algorithm proposed by Krebs et al.^47^ This adaptive challenge approach has been widely implemented in robotic rehabilitation. In this approach, the patient initiates movement and the robot adaptively adjusts force, impedance, and/or game parameters to promote target levels of task success.

### Prevention of Slacking

We have shown that overtly guiding the patient during robotic therapy causes them to slack, which we define as an automatic, slow reduction in effort (or force) when kinematic error is small.^32^ CIMT used a mitt to limit involvement of the good hand. Implicit in this approach is the idea that individuals will find a way to reduce effort if they can. In our own work on slacking, we have identified a computational algorithm the brain uses to reduce effort when task error is small, an algorithm we call “slacking.”^32,48-50^ Rehabilitation robotics must prevent slacking; engagement and effort are essential pre-conditions for promoting use-dependent motor recovery. The only 2 clinical trials I know of where robotic therapy significantly underperformed a comparison therapy are a study of an early version of the Lokomat, in which slacking was not actively addressed,^51^ and a study in which the control group participated in subject-passive, robot-driven, wrist motion.^52^

### Participant Selection

Fifth, it matters who participates. The EXCITE trial required individuals to have 10° of finger extension ability. We showed recently that individuals a small amount of finger function also benefit most from robotic therapy.^34^ They are optimal responders presumably because they have a “weak” cortical spinal tract (CST). We hypothesize that a weak CST is a key residual resource needed for practice-driven improvements in hand movement ability because the brain’s massive parallelism is a primary enabler of beneficial motor plasticity. As we have shown using a computational model of rehabilitation, a massively parallel effector system (the CST, comprised of ~1M neurons when uninjured^53^) that produces forces based on the principle of recruitment (ie, it must recruit thousands of CST neurons to activate muscles) and learns through stochastic search (a form of reinforcement learning) explains multiple features of force recovery after stroke.^54,55^ As an aside, when Steve Wolf learned of our proposal to build computational models of neurorehabilitation,^56^ he didn’t say “That’s silly – too complicated,” but was immediately encouraging and offered to provide longitudinal grip force data from the EXCITE trial to help build models.

Pragmatically, it is extremely unsatisfactory to say that only some people can benefit from movement training (those with unambiguous CST preservation) and leave it there. Thus, my group has sought to invent new forms of technology-assisted movement training that can benefit often-excluded cohorts of more severely impairment individuals, such as individuals where CST preservation is ambiguous. Most recently, we developed a dynamic arm rest called Boost^11^ that stroke inpatients with severe arm paresis can use during “wheelchair time” to practice elbow extension ability early after stroke. We found that individuals can add ~300 movements per day without direct supervision (Figure 1(F)).^57^ As described above, Steve Wolf has also experimented with an impressive diversity of movement training approaches throughout his career, applicable to several different populations. When, I reviewed them for this article, it brought to mind the spirit of a favorite quote from Pulitzer-prize winning author Marilynne Robinson, “What we have expressed, compared with what we have found no way to express, is overwhelmingly the lesser part.”^58^

### Proprioception Predicts Responders

This is more of an emerging result in robotic therapy, but we recently found that individuals with poor finger proprioception, measured robotically with the FINGER robot (Figure 1(G)), did not benefit from intensive robotic hand training.^10^ Damage to somatosensory areas was similarly predictive.^35^ Approximately half of individuals post-stroke have finger proprioception deficits, a substantial population.^59^ When I presented this result at a conference Steve was attending, he alerted me to a retrospective study of the EXCITE trial that showed that individuals with impaired proprioception at baseline had only a 20% probability of achieving a clinically meaningful outcome compared with those with intact proprioception.^60^ This is a sixth parallel between CIMT and rehabilitation robotics (Figure 2).

Mechanistically, these proprioception results fit with our theoretical model framing stroke recovery as a stochastic search: the search needs a teaching signal.^54,55^ Without a clean proprioceptive teaching signal, we postulate that the search for viable fragments of the CST to use to drive motoneuronal pool recruitment becomes inefficient or even impossible. Alternately, intact proprioception might also be key within a Hebbian framework, in which neural pathways are reinforced through repeated co-activation of sensory and motor signals.^10^

## Implications Arising From This Work for Future Directions in Neurorehabilitation

Finally, I will discuss 5 implications for future directions in robotic therapy, relating them to Dr. Wolf’s contributions and vision.

### The Use of Shared Principles From CIMT and Robot-Assisted Therapy to Guide the Design of Advanced Rehabilitation Technologies

The first implication is that the conceptual synergies between CIMT and Robot-Assisted Therapy – including the principles of dose, task-specific training, shaping, prevention of slacking, participant selection, and the critical role of proprioception – provide a powerful conceptual foundation for anyone interested in the design and expression of new rehabilitation technologies. When I started working in the field, it was unclear what a robotic therapy device should do. To those starting now, these principles are an excellent starting framework.

### Precision Rehabilitation That Prioritizes Proprioception

Second, there is a need for precision rehabilitation.^61^ Robotic rehabilitation, like all existing forms of movement rehabilitation, produces modestly beneficial reductions in motor impairment on average, but the variance is nearly as high as the mean benefit across participants. I estimate that roughly 30% benefit by a clinically significant amount.^34^ Progress in robotic rehabilitation, and rehabilitation in general, requires replacing uniform protocols with tailored treatments that account for patient-specific recovery differences. This perspective aligns with broader efforts to define precision approaches in stroke recovery, including the Stroke Recovery and Rehabilitation Roundtable, to which Steve contributed, which emphasized the need for standardized biomarkers, stratification of patients, and more targeted interventions.^62^

While progress is being made in identifying brain-based biomarkers for predicting recovery,^63^ my laboratory is pursuing the idea that precision rehabilitation should consider the sensory side of sensory motor control – especially proprioception, for the reasons described in the last section – that is proprioception predicts movement training response because it is a key “teaching signal.” Promisingly, proprioceptive ability is learnable,^64^ showing several of the same features as motor learning, including specificity, 2 time constant acquisition, retention, and transfer.^65^ Yet, despite the clinical evidence and theoretical basis for the importance of proprioceptive deficits post-stroke, and the fact that proprioception is trainable, therapists rarely quantitatively assess it^66^ and there are few methods routinely used to target proprioceptive deficits in clinical stroke rehabilitation practice.

Steve’s work is again inspiring here. CIMT therapy flowed out of research on deafferented non-human primates that showed that eliminating only the *sensory* side of sensory-motor control, not the motor side, caused animals to stop using their limb, similar to individuals post-stroke with hemiparesis (“learned non-use”).^67^ Steve’s review of the potential therapeutic mechanisms of Tai Chi for balance improvement highlights that a basic principle of Tai Chi is awareness of presence and movement of the body within its own space; that is proprioceptive attention.^68^ His work on the VibroTactile Stimulation (﻿VTS) glove, a device that delivers vibration to the affected hand for multiple hours per day, is based on the assumption that somatosensory stimulation will promote motor recovery.^44^ Finally, the Assisted Movement with Proprioceptive Stimulation (AMPS) trial, which Steve contributed to, examined a robotic training technique that focused on proprioceptive stimulation through vibration of antagonist muscles.^69^ This study found that AMPS training was about twice as effective at reducing hand impairment as a matched amount of conventional therapy.

In standard robotic training, movement training is visually guided through gamified computer interfaces. To more directly target and challenge proprioception ability during training, we recently developed a proprioceptive gaming approach called “Propriopixels,” which replaces select visual gaming elements with proprioceptive cues delivered by the robot by moving the hand.^42,70^ This approach requires individuals to intensively focus attention on proprioceptive input during game play. This is a new way to play video games – by combining sight and “proprioceptive feel.” We recently found that this approach was motivating and feasible even for individuals with impaired finger proprioception.^42^ The results of a recent clinical trial we conducted with chronic individuals post-stroke suggest that, for individuals with impaired proprioception, this proprioceptively-focused form of robotic training was more effective at improving hand function than standard, visually-driven robotic therapy. This brings us back to precision rehabilitation: if presence of residual CST function can help identify candidates for movement training, perhaps impairment in baseline proprioceptive function can identify individuals who would benefit from 2 fundamentally different types of movement training: motor- versus proprioceptively- focused training.

### The Design of Practical, Widely Adoptable Robotic Systems

Third, there is a need for practical robots. While used intensively in some clinical environments, robotic therapy devices have struggled to gain widespread uptake.^71,72^ As it stands, we believe that delivering engaging therapy early and intensively matters more than device complexity. If so, an important question is what forms of technology-assisted training are more likely to be adopted, and which are more likely to encourage long-term perseverance with training. We believe that designing technology that is exceptionally easy-to-use is critical.^73^ We have also generated experimental evidence that shows that technology should provide customized training experiences at appropriate success levels to encourage long-term use.^74,75^ Engineers need to work with clinicians and patients to make devices simpler to use and easier to keep using. We also anticipate that behavioral coaching methods, implemented through episodic remote patient monitoring and/or artificial intelligence, will also make a difference, similar to the way coaching is a key part of CIMT. The point is to develop an accessible toolbox of technology that a person post-stroke can use to safely and effectively continue their therapy without requiring continuous one-on-one interactions with skilled therapists, moving rehabilitation dose 1 step closer to aspirational levels. Consistent with these concepts, recent work involving Steve has highlighted that stroke survivors identify ease of use, perceived benefit, and integration into daily routines as key determinants of adoption of rehabilitation technologies.^76^

### The Development of Real-Time Biomarkers and Closed-Loop Training Systems That Optimize Recovery

**Figure 3.**
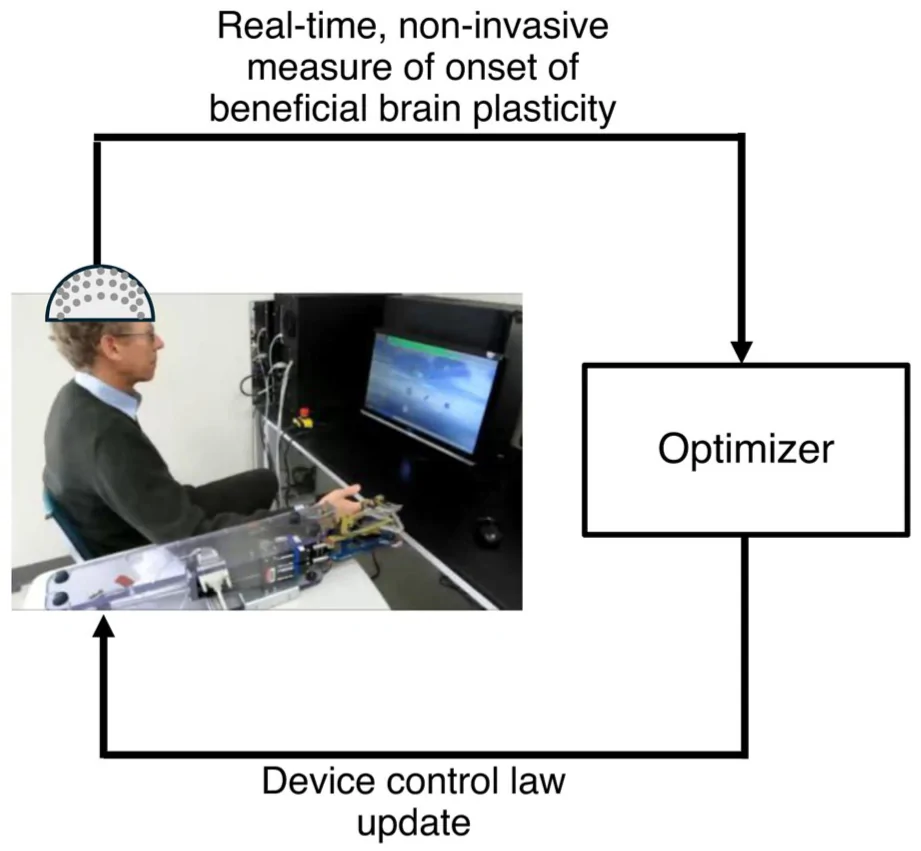
A moonshot technology for robotic rehabilitation: a real-time, non-invasive measure of the onset of beneficial brain plasticity that can be leveraged to perform human-in-the-loop optimization^77^ of the human-robot interaction, in order to optimally drive plasticity.

Fourth, considering now more of a moonshot technology, it would be immensely helpful to have a short-term biomarker of beneficial long-term plasticity that could be leveraged by a closed-loop training system. Rehabilitation science, including robotic rehabilitation, suffers from the cost and time needed to vet each new intervention or device in a clinical trial. If one could put a non-invasive sensor over a person’s brain and optimize the intervention in real-time (Figure 3), it would accelerate progress. This approach has been achieved for energy minimization during walking using wearable robotic assistance, an approach called “human-in-the-loop optimization.”^77^ In this case, changes in V02 are measured on the timescale of minutes, providing a means to make real-time technology adjustments to minimize energy consumption. We need human-in-the-loop optimization for robotic movement training, enabled by a new sort of “Brain02” – a real-time brain biomarker predicting the early onset of future, beneficial, synaptic plasticity. Even better, if we could accurately model motor recovery, we could optimize robotic training paradigms by learning through simulations,^56^ an approach that also recently achieved success in the field of energy minimization during walking using exoskeletons.^78^ This perspective again aligns with the recommendations Steve contributed to in the Stroke Recovery and Rehabilitation Roundtable – that is standardized biomarkers, stratification of patients, and more targeted interventions – but integrates them in a dynamic, algorithmic methodology that leverages mathematical optimization.

### The Need for Deeper Collaboration With People With Lived Experience of Disability

Fifth, returning to Marilynne Robinson’s concept that what we have yet to imagine far exceeds what we have so far imagined, rehabilitation robotics needs to diversify beyond retraining hand and leg control of individuals post-stroke with minimal cognitive impairment. There is a place for a Monet Haystack Painting Approach to address a focused problem. Monet painted 25 haystack paintings in a 2-year period, an analogy I return to when reflecting on the many iterative solutions we’ve explored for upper extremity movement training (eg, Figure 1). However, instead of continuing to go deeper with a single problem, there is also a need to expand the focus of rehabilitation robotics, as James Sulzer has written persuasively about following the traumatic brain injury of his 3 year old daughter.^79^ He highlights how existing technologies fail to meet real needs. I recently experienced the enormity of unsolved problems in rehabilitation engineering caring for my mother, who was healthy but died from complications from a fall, and my father, who died from complications from Alzheimer’s disease. It seems to me that rehabilitation robotics and other related technologies could play a role in many more unsolved problems, with fall prevention and care of individuals with cognitive impairment being 2 of the most pressing and impactful. New types of muscle-like actuators, coupled with sensor-driven artificial intelligence, could help drive breakthroughs in both wearables for fall prevention and socio-physical assistance for individuals with cognitive impairment. The history of CIMT can serve as a model for the diversification robotic rehabilitation, as CIMT has evolved into a translational body of work spanning basic science, clinical trials, and diverse patient populations, extending well beyond the original EXCITE trial.^80^

## Conclusion

As Sulzer advocates, tightly incorporating the expertise of individuals with lived experience of disability at all stages and in all facets of research, and sufficiently funding their insights, will help identify other important, unsolved problems and generate new, effective solutions. Steve Wolf operationalized lived experience via patient-chosen goals and patient-reported outcome measures embedded in large multi-site trials. Further, the EXCITE trial stands as one of the most influential rehabilitation trials of the past several decades, fundamentally reshaping expectations for recovery after stroke and establishing key principles that continue to guide the field. Much of the work described here, including advances in robotic therapy and proprioception-targeted training, builds on and reinforces this conceptual foundation. Importantly, Steve’s work consistently extended beyond any single intervention, reflecting an early and sustained openness to emerging technologies and approaches – from constraint-induced therapy to robotics, wearable sensing, stimulation, and community-based frameworks for recovery. This is the model the robotic therapy field needs to adopt as it continues to progress. Steve has advocated for the types of interdisciplinary collaborations that are critical for advancing the robotic therapy field, and he has exemplified the listening and collaborating skills required. In doing so he helped redefine what rehabilitation science could achieve. His impact on robotic therapy and the broader field of rehabilitative movement training is both lasting and still unfolding.
